# Is Preoperative Antimicrobial Prophylaxis Necessary in Testicular Torsion Surgery? Results from the National Surgical Quality Improvement Program Pediatric

**DOI:** 10.1055/a-2786-3629

**Published:** 2026-01-22

**Authors:** Alexandra Stone, Maithili Gopalakrishnan, Anthony Tracey, Matthew Mason, Jeffrey Villanueva

**Affiliations:** Department of Urology, SUNY Upstate Medical University, Syracuse, New York, United States

**Keywords:** antibiotics, prophylaxis, testicular torsion

## Abstract

**Objective:**

This study aimed to evaluate the impact of surgical antimicrobial prophylaxis (SAP) on testicular torsion surgery (TTS) postoperative outcomes using data from the National Surgical Quality Improvement Program Pediatric (NSQIP-P). Across multiple studies, NSQIP-P has proven increased sensitivity in recording postoperative complications compared with similar databases.

**Methods:**

The 2021–2023 NSQIP-P participant user and SAP files were queried for all TTSs. Patients with unrecorded SAP administration data ( *n* = 2,725) were excluded. Postoperative events were then compared between subjects who did or did not receive SAP. Primary outcomes included rates of surgical site infection (SSI), 30-day readmission, and 30-day reoperation. SPSS statistical software was used to perform comparative statistical analyses between groups.

**Results:**

A total of 614 patients were included in the study and divided into Group 1 (+SAP, *n* = 322) and Group 2 (−SAP, *n* = 292). In the +SAP group, there was no observation of SSI, but one case resulted in 30-day readmission and reoperation. Another 30-day readmission and 30-day reoperation were noted, as well. In the −SAP, there was one observation of a deep incisional SSI who was readmitted. There were no 30-day reoperations in this group. There was no statistical significance in outcomes between the two groups.

**Conclusion:**

To date, this is the first study assessing the impact of SAP in torsion-reduction surgeries using the NSQIP-P database. There is a low frequency of postoperative complications with this procedure. Our study suggests limited utility of SAP with this surgery despite continued use.

## Introduction

Testicular torsion is the twisting of the spermatic cord that affects 3.8 per 100,000 young males and is a surgical emergency requiring immediate intervention. This can include orchiopexy if the testicle is deemed viable and orchiectomy in cases where the testicle has already necrosed. 1 Current antibiotic prophylaxis guidelines for pediatric orchiopexies indicated for testicular torsion vary widely among different health care centers. Providers often rely on previous experience or the balance of antibiotic benefits and harms to determine the prescription necessity of pediatric subspecialty surgeries. 2 Optimization of intraoperative antibiotic prophylaxis is warranted as current standardization procedures are insufficient. There is an evident risk associated with an arbitrary prescription. With the increasing prevalence of antibiotic stewardship, it is important to challenge the efficacy of surgical antimicrobial prophylaxis (SAP). 3

The American College of Surgeons (ACS) National Surgical Quality Improvement Program Pediatric (NSQIP-P) allows health care centers to report aspects of surgical cases that can be trended to help improve clinical quality. Systematic review of research centers comparing NSQIP-P to their own hospital databases uncovered that the database allows multi-institutional comparison and larger generalizability. NSQIP-P sensitivity to surgical site infections (SSIs) was similar, if not more advanced than, many of the control databases. 4 5 Across multiple studies, the NSQIP-P database has been shown to have increased sensitivity in recording postoperative infections and complications compared with similar databases. 6 7 8 9 It is widely accepted as a reliable tool to measure various outcomes following surgery.

We hypothesize that within the NSQIP-P database, there will be no association between SAP use and rates of SSIs or wound complications despite the persistent prevalence of SAP use in testicular torsion surgeries.

## Methods

### Study Design and Data Source

We used data from the 2021–2023 ACS NSQIP-P database to conduct a retrospective cross-sectional study among pediatric patients undergoing torsion reduction surgery.

The ACS NSQIP-P database is a nationally validated, risk-adjusted, outcomes-based approach to measure and improve surgical care for pediatric patients. The database is currently used by 60 hospitals to make informed decisions about improving the quality of care. The data from NSQIP-P are collected and reviewed by trained abstractors rather than via claims data, making it a reliable and standardized source. Additionally, NSQIP-P is specifically designed to take into consideration the low morbidity and mortality rates of pediatric surgical procedures, restricting certain operations in its sampling plan. 10

### Inclusion Criteria and Study Variables

We queried the NSQIP-P participant user files to include all pediatric patients who underwent reduction of testicular torsion in 2021, 2022, and 2023 ( *n* = 3,339). Patients with missing information regarding SAP administration ( *n* = 2,725) were excluded from the study.

### End Points

We sought to evaluate the impact of receiving SAP. Our primary outcomes of interest were rates of SSIs, readmission, and reoperation within 30 days of torsion surgery. Secondary outcomes of interest included superficial SSIs, deep SSIs, and urinary tract infections.

### Predictor of Interest

Exposure of interest was the administration of a single dose of preoperative intravenous antibiotics. We evaluated the association of preoperative antimicrobial prophylaxis with postoperative outcomes.

### Covariates

Various demographic and clinical variables were collected as covariates to include in the analysis. These variables included age (in years), race (White, Black, Asian, or Other), prematurity status (yes, no, or unknown), American Society of Anesthesiologists (ASA) score (1, 2, or 3), and operative time (in minutes).

### Statistical Analysis

Statistical analyses were performed using IBM SPSS Statistical Software 26.0.

Continuous variables were analyzed using an independent samples *t* -test (Mann–Whitney U) and were recorded with mean and standard deviation. Categorical variables were analyzed using chi-square analysis and were recorded as proportions. Multivariable binomial logistic regression was used to evaluate odds ratios and 95% confidence intervals of the association between SAP redosing and our primary outcomes, rates of SSIs and complications. Multivariate analysis adjusted for various a priori selected covariates such as age (in years), race (White, Black, Asian, or Other), prematurity status (yes, no, or unknown), ASA score (1, 2, or 3), and operative time (in minutes). All statistical tests were two-tailed, with *p* -values <0.05 being considered statistically significant.

## Results

A total of 614 patients were included in the study, as shown in Table 1 . The majority of patients received SAP. The cohort was predominantly White and born full term. The patients who received antimicrobial prophylaxis were overall slightly older than those who did not. Patients who received SAP tended to have lower ASA scores with shorter operative times.

**Table 1:** Comparative analysis between patients who received SAP (+SAP) and those who did not (−SAP) prior to testicular torsion surgery

	+SAP ( *N* = 322)	−SAP ( *N* = 292)	*p* -Value
Age (years)	14.0 (IQR 12.3–15.5)	13.9 (IQR 12.5–15.2)	0.285
Race			0.402
White	171 (53.1%)	150 (51.3%)	–
Asian	18 (5.6%)	9 (3.1%)	
Black	48 (14.9%)	47 (16.1%)	
Other	85 (26.4%)	86 (29.5%)	
Prematurity			0.230
No	237 (73.6%)	194 (66.4%)	–
Yes	12 (3.7%)	16 (5.5%)		
Unknown	73 (22.7%)	82 (28.1%)		
ASA status			0.313
ASA 1	211 (65.5%)	174 (59.6%)	
ASA 2	104 (32.3%)	111 (38.0%)	
ASA 3	7 (2.2%)	7 (2.4%)	
Operative time (minutes)	34.0 (IQR 28.0–46.0)	43.0 (IQR 34.0–53.0)	0.969
Surgical site infections	0	1	0.293
30-Day readmissions	2	1	0.621
30-Day reoperations	2	0	0.177

Abbreviations: IQR, interquartile range; SAP, surgical antimicrobial prophylaxis.

Among the group that did not receive SAP, there was one SSI, whereas there were no SSIs in those who received SAP. When comparing 30-day outcomes, there were more 30-day readmissions and reoperations among the patients who received SAP as opposed to those who did not. Neither of these differences was statistically significant. As shown in Table 2 , one of the readmissions was due to wound complications, more specifically, a deep incisional SSI in a patient who did not receive SAP. The other readmissions were among patients who received cefazolin, one due to bleeding and the other a renal injury. There was a reoperation in the SAP cohort that did not result in readmission, an incision and drainage for a non-inflammatory condition of the testis, 1 day after the initial operation.

**Table 2:** Data for readmission and reoperation events

Patient number	1	2	3	4
SAP administration	Cefazolin	No	Cefazolin	Cefazolin
ASA score	1	3	1	2
Readmission	Yes	Yes	Yes	No
Reoperation	No	No	Yes	Yes
Event description	Renal injury	Deep incisional SSI	Bleeding	Incision and drainage
Days from initial operation	4	7	1	1

Abbreviations: SAP, surgical antimicrobial prophylaxis; SSI, surgical site infection.

## Discussion

Current practice for pediatric urologic procedures does not follow specific antibiotic stewardship guidelines, as there is no agreement on appropriate usage. Often, surgeons will follow institutional practice patterns, which are also not cohesive among hospitals. Past studies have attempted to extract the most prevalent risk factors for SSI, UTI, or bacteremia to determine when antibiotic prophylaxis is warranted. Due to the limited number of studies and wide variability among results, there is no clear consensus. 3 11 This lack of conclusive evidence carries over to pediatric surgeries, where the variability is even more extreme as children have ever-changing genitourinary systems from infancy to adolescence. This leads many physicians to practice personal or institutional preference, further increasing heterogeneity in prescription patterns.

With haphazard usage of antibiotics comes a large burden on the health care system. A retrospective study spanning 890 hospitals across the United States investigated the rates of hospitalizations following hospital and community-acquired infection with antibiotic-resistant bacteria. It was concluded that these infections composed more than 20% of U.S. hospitalizations during the period (2012–2017) in which the study was conducted. 12 This study highlights that improper usage of antibiotics places a burden on patient health, health care workers, and health care systems. With the increasing prevalence of antibiotic resistance, there comes a growing need for antibiotic stewardship, especially among pediatric urologic surgeries, in which there is no clear consensus. The data remain unclear on whether SAP helps diminish infection rates. One study showed that children who received antibiotic prophylaxis had significantly elevated chances of developing *Clostridioides difficile* infection as compared with those who did not receive SAP. 13 The risk of colonization or infection with *C. difficile* following antimicrobial prophylaxis is poorly understood among clinicians.

Additionally, in our study, bleeding was found to be one of the causes of readmission and reoperation events. Among these two patients, the +SAP group received cefazolin as their preoperative antibiotic. These are not isolated incidents. A 2024 study highlights the increased risk of bleeding in patients treated with cefazolin. At least one clinically relevant non-major bleeding event or one major bleeding event was seen in 10% of patients treated with cefazolin in their sample. 14 Another study from 2019 correlates five severe hemorrhage events with cefazolin treatment. 15 There is increasing awareness of bleeding events following cefazolin treatment, further highlighting the need to standardize the use of SAP in urologic procedures. Another complication causing readmission was renal injury. While there is no concrete evidence for an established correlation between cefazolin use and acute kidney injury, there are some studies highlighting renal injury in combination therapy of cefazolin and gentamicin or vancomycin. 16 17 A case report from 2018 calls attention to the potential for acute interstitial nephritis in the setting of cefazolin use. Oftentimes, patients will have minimal symptoms; the patient in this case had nephrotic-range proteinuria, which was worked up. 18 Given that cefazolin is renally excreted, dosage adjustments are recommended in pediatric and adult patients with renal impairment, further highlighting the relationship between the drug and renal function. 19

Despite this discordance, there is a persistent prevalence of SAP use among physicians. While prior literature has investigated the impact of SAP on postoperative complications following other urologic surgeries such as hypospadias repair, kidney transplantation, and circumcision, the impact on testicular torsion surgeries has not been studied. 3 20 A 2022 study conducted in Italy featured recommendations for clean urologic procedure antibiotic prophylaxis based on retrospective data collection. SAP and SSI risk were found to have no associations for clean procedures such as circumcision, circumcision revision, or penile torsion repairs. For clean-contaminated procedures, defined as any opening into the genitourinary tract, such as hypospadias repair, it is recommended to administer preoperative and postoperative prophylaxis depending on the specific procedure. 3 Our study shows that SAP has no impact on postoperative complications after testicular torsion surgery.

Our study has several limitations. This study uses a database containing retrospectively collected data, which is susceptible to selection bias. Although the database included over 3,000 cases of testicular torsion, only 614 contained SAP administration information, which decreased the overall power of the study. While we would prefer to have a more substantial sample size, testicular torsion is a rare event that affects such a small population of young males, and any dedicated, prospective study would be difficult to complete. Additionally, we were unable to extract information from the database to separate the cases by bilateral orchiopexy versus unilateral orchiectomy, which would have yielded a more complete analysis of the confounders that may contribute to postoperative infection rates. However, given that we saw very few infections in our study populations, it would likely not have changed the overall results showing that SAP use had no significant impact on postoperative complications.

## Conclusion

Despite the prevalence of SAP use in torsion reduction surgeries, our study finds that SAP use is not significantly associated with the postoperative rate of wound infections, readmission, and reoperation. Our findings suggest that it would be appropriate in clinical practice to forgo SAP use in torsion reduction surgeries.
